# Astaxanthin supplementation counters exercise-induced decreases in immune-related plasma proteins

**DOI:** 10.3389/fnut.2023.1143385

**Published:** 2023-03-21

**Authors:** David C. Nieman, Jongmin Woo, Camila A. Sakaguchi, Ashraf M. Omar, Yang Tang, Kierstin Davis, Alessandra Pecorelli, Giuseppe Valacchi, Qibin Zhang

**Affiliations:** ^1^Human Performance Laboratory, Appalachian State University, Kannapolis, NC, United States; ^2^UNCG Center for Translational Biomedical Research, University of North Carolina at Greensboro, Kannapolis, NC, United States; ^3^Department of Food Bioprocessing and Nutrition Sciences, Plants for Human Health Institute, North Carolina State University, Kannapolis, NC, United States; ^4^Department of Environmental Sciences and Prevention, University of Ferrara, Ferrara, Italy

**Keywords:** astaxanthin, exercise, proteomics, oxylipins, inflammation, cytokines

## Abstract

**Objectives:**

Astaxanthin is a dark red keto-carotenoid found in aquatic animals such as salmon and shrimp, and algae (Haematococcus pluvialis). Astaxanthin has a unique molecular structure that may facilitate anti-oxidative, immunomodulatory, and anti-inflammatory effects during physiological stress. The primary objective of this study was to examine the efficacy of 4-week ingestion of astaxanthin in moderating exercise-induced inflammation and immune dysfunction using a multi-omics approach.

**Methods:**

This study employed a randomized, double blind, placebo controlled, crossover design with two 4-week supplementation periods and a 2-week washout period. Study participants were randomized to astaxanthin and placebo trials, with supplements ingested daily for 4 weeks prior to running 2.25 h at close to 70%VO_2max_ (including 30 min of 10% downhill running). After the washout period, participants repeated all procedures using the counterbalanced supplement. The astaxanthin capsule contained 8 mg of algae astaxanthin. Six blood samples were collected before and after supplementation (overnight fasted state), immediately post-exercise, and at 1.5, 3, and 24 h-post-exercise. Plasma aliquots were assayed using untargeted proteomics, and targeted oxylipin and cytokine panels.

**Results:**

The 2.25 h running bout induced significant muscle soreness, muscle damage, and inflammation. Astaxanthin supplementation had no effect on exercise-induced muscle soreness, muscle damage, and increases in six plasma cytokines and 42 oxylipins. Notably, astaxanthin supplementation countered exercise-induced decreases in 82 plasma proteins (during 24 h recovery). Biological process analysis revealed that most of these proteins were involved in immune-related functions such as defense responses, complement activation, and humoral immune system responses. Twenty plasma immunoglobulins were identified that differed significantly between the astaxanthin and placebo trials. Plasma levels of IgM decreased significantly post-exercise but recovered after the 24 h post-exercise recovery period in the astaxanthin but not the placebo trial.

**Discussion:**

These data support that 4-week astaxanthin versus placebo supplementation did not counter exercise-induced increases in plasma cytokines and oxylipins but was linked to normalization of post-exercise plasma levels of numerous immune-related proteins including immunoglobulins within 24 h. Short-term astaxanthin supplementation (8 mg/day during a 4-week period) provided immune support for runners engaging in a vigorous 2.25 h running bout and uniquely countered decreases in plasma immunoglobulin levels.

## Introduction

Athletes experience recurrent training and competitive increases in inflammation, oxidative stress, and immune dysfunction (1). Nutrition-based strategies including the recent emphasis on increased intake of plant phytochemicals are being explored as countermeasures to exercise-induced physiological stress (2). A recent focus in our research group has been the use of metabolomics, lipidomics, and proteomics to capture the complex responses from nutrition interventions within an exercise context (3–7).

The primary objective of this study was to examine the efficacy of 4-week ingestion of the keto-carotenoid astaxanthin in moderating exercise-induced inflammation and immune dysfunction. Astaxanthin is a dark red carotenoid found in aquatic animals such as salmon and shrimp. Humans cannot synthesize astaxanthin and can only acquire it through their diet. In the dietary supplement industry, natural astaxanthin is extracted from algae (Haematococcus pluvialis). Astaxanthin lacks pro-vitamin A activity but is more bioactive than other carotenoids such as zeaxanthin, lutein, and carotene in exerting anti-oxidative, immunomodulatory, and anti-inflammatory effects (8, 9).

Astaxanthin has a unique molecular structure with a high capacity to scavenge reactive oxygen nitrogen species (RONS) and other reactive species (sulfur and carbon) directly by donating electrons and bonding with the free radical to form a non-reactive product (8, 9). Astaxanthin may protect muscle cell membranes in salmon during their long migrations, and this finding prompted rodent-based studies that showed attenuation of exercise-induced damage in skeletal and heart muscle (10, 11). Astaxanthin plays a regulatory role with transcription factors involved in cellular redox homeostasis and inflammation including nuclear-factor erythroid 2-related factor 2 (Nrf2) and nuclear factor κB (NF-κB), respectively (12). In addition, astaxanthin may exert immune-regulatory effects by augmenting immunoglobulin production and enhancing natural killer and T lymphocyte responses (12–14).

Taken together, these data suggest that astaxanthin has the potential to mitigate post-exercise oxinflammation and immune dysfunction (2, 7). Few high quality randomized controlled trials utilizing prolonged and intensive exercise bouts have been published, and these studies used disparate dosing regimens, varied research designs, and limited outcome measures that provided inconclusive findings (15–20). To better assess the potential influence of astaxanthin supplementation on exercise-induced physiological stress, this study emphasized a multi-omics approach (4, 7). Oxylipins are bioactive oxidation products generated during stressful exercise from the metabolism of *n*-6 and *n*-3 polyunsaturated fatty acids (PUFAs) by cyclooxygenase (COX), lipoxygenase (LOX), and cytochrome P450 (CYP) enzyme systems. Oxylipin generation during exercise can be moderated through nutritional interventions (1, 2, 5–7, 21). Untargeted proteomics is the large-scale study of the proteome or the entire set of proteins produced in response to a wide variety of stresses (7). The utilization of untargeted proteomics in human sports nutrition studies is an emerging science and has high potential to improve scientific understanding regarding the complex interplay between exercise-and nutrition-related influences on immune and metabolic responses (7, 22, 23).

## Methods

### Study participants

**Figure 1:**
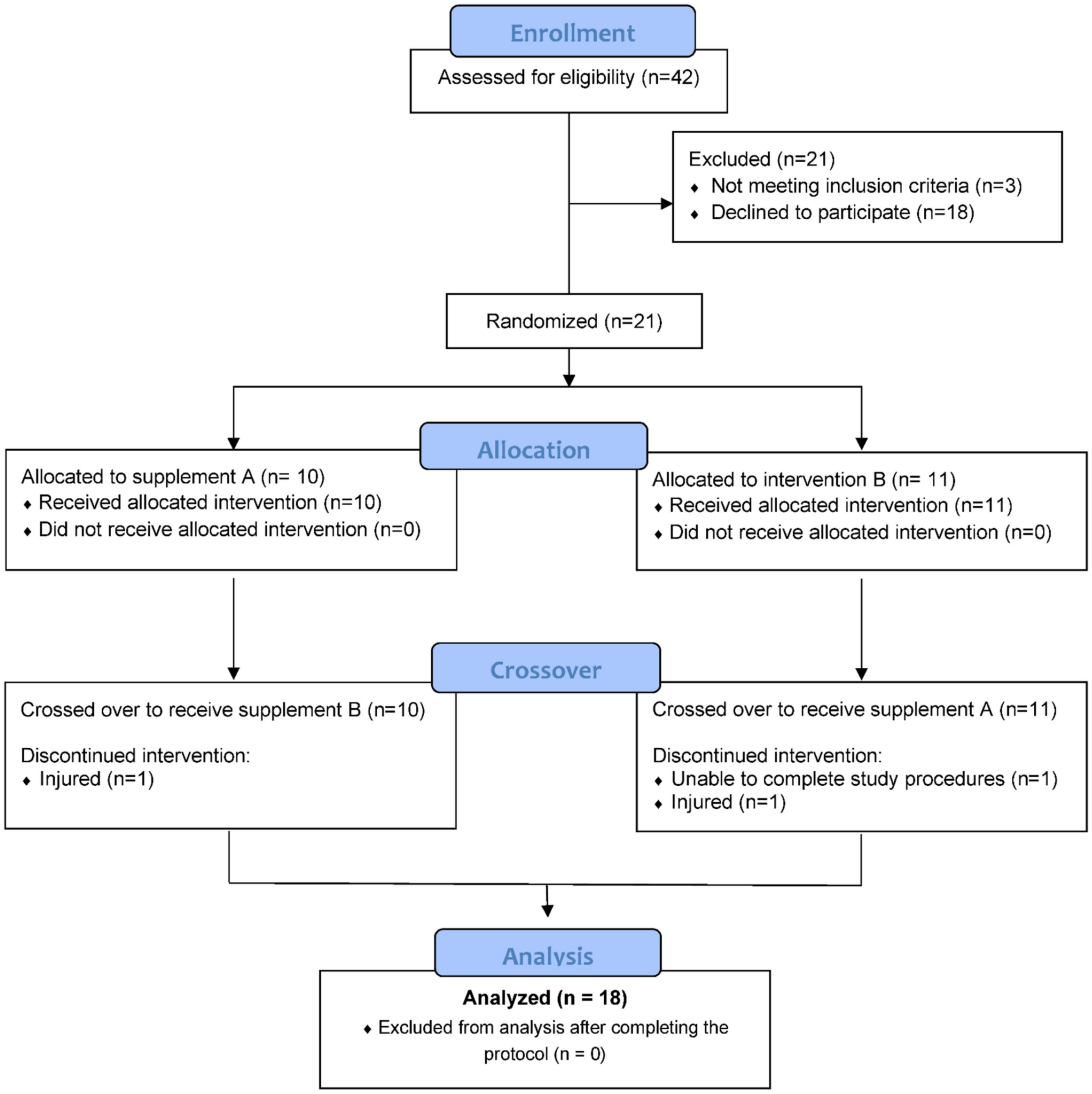
Study participant flow diagram.

Healthy, non-smoking male and female runners were invited to take part in this study if they met the inclusion criteria including 18–57 years of age, capable of running 2.25 h on laboratory treadmills at 70% maximal oxygen consumption rate (VO_2max_), and a willingness to avoid supplements and medications such as non-steroidal anti-inflammatory drugs (NSAIDs) with a potential to influence inflammation and immune function. Participants also agreed to avoid foods and supplements with astaxanthin during the 10-week study (other than what was provided) including algae, yeast, salmon, trout, krill, shrimp, and crayfish. After 42 participants were assessed for eligibility, 21 were entered into the study, with 18 completing the protocol (Figure 1). The study participant number provided more than 84% power to detect a difference with an effect size 0.7 at alpha 0.05 using two-sided *t*-tests. Participants voluntarily signed the informed consent, and procedures were approved by the university’s Institutional Review Board. Trial Registration: ClinicalTrials.gov, U.S. National Institutes of Health, identifier: NCT05409092.

### Study design

**Figure 2:**
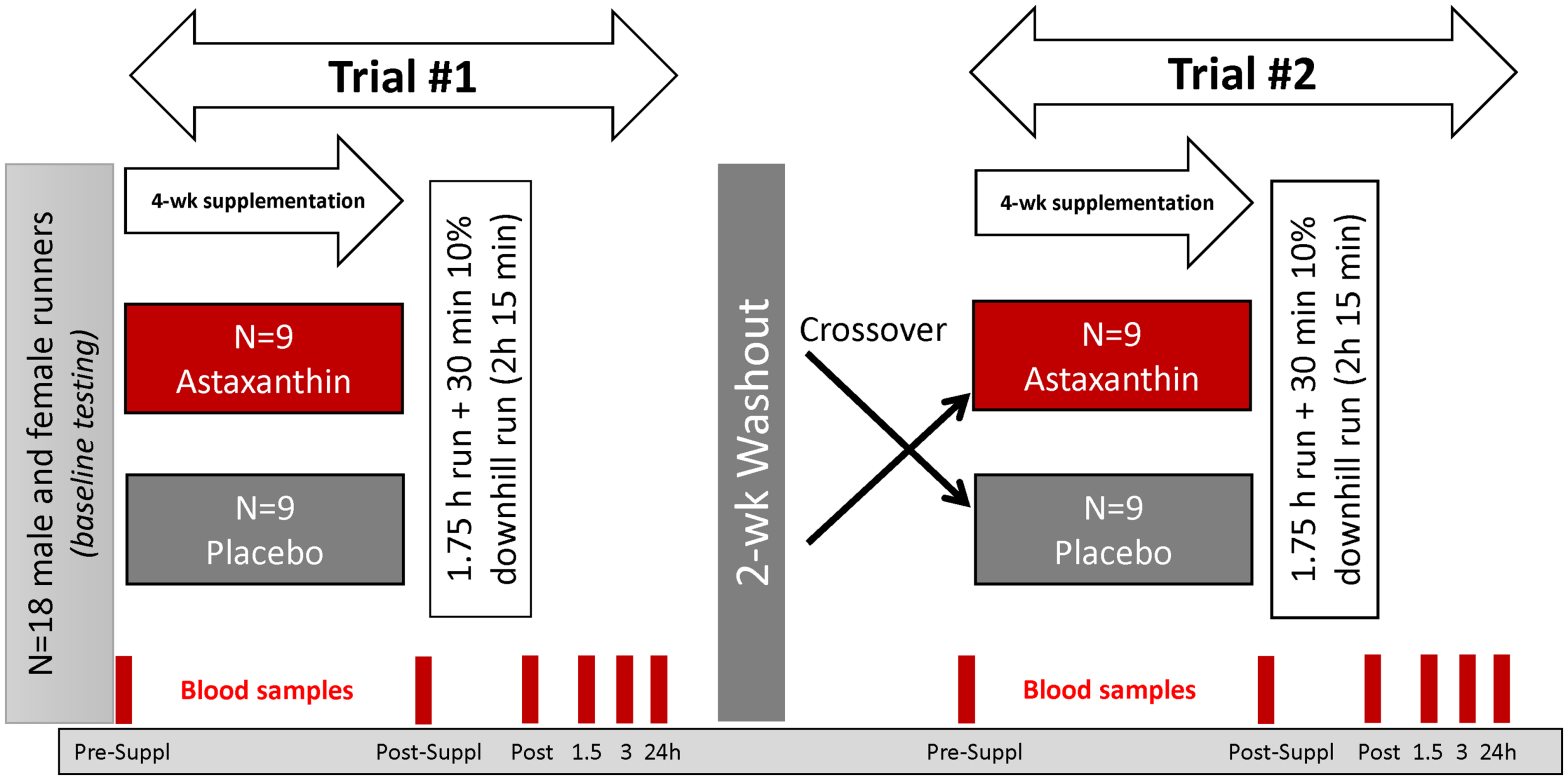
Study design.

This study employed a randomized, double blind, placebo controlled, crossover design with two 4-week supplementation periods and a 2-week washout period (Figure 2). The study included seven lab visits at the Appalachian State University Human Performance Laboratory (HPL) at the North Carolina Research Campus, Kannapolis, NC.

Study participants were randomized to astaxanthin and placebo trials, with supplements ingested daily (with the first meal) for 4-week prior to participation in the first 2.25 h running session. After a 2-week washout period, participants repeated all procedures using the counterbalanced supplement. The astaxanthin and placebo supplements were supplied by the sponsor (Lycored, Be′er Sheva, Israel). The astaxanthin capsule contained 8 mg of astaxanthin from algae in starch beadlets, and the placebo capsules contained the starch beadlets without astaxanthin. The astaxanthin and placebo capsules were identical in appearance and color. Four ounces (113 g) of sockeye salmon contains about 4.5 mg of astaxanthin, with much higher levels in arctic shrimp and krill. Natural astaxanthin is sold around the world as an antioxidant supplement with a recommended dosage of 4–12 mg a day. A 4-week supplementation period with 8 mg/day astaxanthin has been shown to significantly increase plasma astaxanthin in human subjects (13). Lycored uses astaxanthin from Haematococcus pluvialis and the family Haematococcaceae.

Six blood samples were collected before and after 4-week supplementation (overnight fasted state, before 8:00 am in the morning), immediately post-exercise, and at 1.5, 3, and 24 h-post-exercise. Blood samples were aliquoted and stored at −80°C prior to analysis for the outcome measures. After each of the blood draws, participants provided a muscle soreness rating using a 1–10 scale (DOMS) (24).

Study participants signed the consent form and were given a complete orientation to the study protocol during the first lab visit. Study participants provided an overnight fasted blood sample and recorded responses to the DOMS questionnaire. Height and body weight were assessed, with body composition measured using the BodPod system (Cosmed, Rome, Italy). Study participants were tested for VO_2max_ during a graded, treadmill test with the Cosmed CPET metabolic cart (Cosmed, Rome, Italy). Supplements for the first and second 4-week supplementation periods were supplied in coded bottles. To facilitate compliance to the supplementation protocol, study participants were contacted *via* email on a regular basis and returned the coded bottles. Participants reported 100% compliance with the supplementation regimen and no adverse events were recorded.

During the 3-day period prior to the 2.25 h running session, subjects tapered exercise training and ingested a moderate-carbohydrate diet using a food list restricting high fat foods, visible fats, and astaxanthin. Participants recorded all food and beverage intake for 3 days at the end of the 4-week supplementation periods to assess the background diet. Macro-and micro-nutrient intake was assessed using the Food Processor dietary analysis software system (Version 11.11, ESHA Research, Salem, OR, United States).

Study participants reported to the Human Performance Lab in an overnight fasted state, provided a blood sample, ingested the astaxanthin or the placebo supplement with water, and then ran 2.25 h at high intensity (70% VO_2max_) while ingesting water alone (3 mL/kg every 15 min). Oxygen consumption carbon dioxide production, respiratory exchange ratio, and ventilation were measured using the Cosmed Quark CPET metabolic cart after 15 min and then every 30 min. Subjects ran 1.75 h followed by 30 min of downhill running (10%) at the same intensity. Blood samples were collected at 0, 1.5, 3, and 24 h post-exercise. Immediately after the 1.5 h post-exercise blood sample, all subjects consumed 8 kcal per kilogram of body weight of a fortified nutrient beverage (Boost, Nestlé S.A., Vevey, Switzerland).

### Sample analysis

Serum creatine kinase and myoglobin, plasma cortisol, and complete blood counts with a white blood cell differential count were analyzed each day samples were collected using Labcorp services (Burlington, NC). Plasma aliquots were prepared and stored in a − 80°C freezer until analysis for cytokines, proteomics, and oxylipins after the study was completed.

#### Plasma cytokines

IL-6, IL-8, IL-10, IL-1ra, monocyte chemotactic protein (MCP-1), and granulocyte colony-stimulating factor (GCSF) from plasma aliquots were measured with the multiplexed immunoassay platform –Ella™ (Protein Simple, CA) (25). Briefly, each individual sample was diluted 2-fold and 50 μL of the diluted sample was loaded to each well on the 32-sample cartridge or 72-sample cartridge, and the concentration of each cytokine was determined with built-in calibration curves. For quality control purposes and measurement reproducibility, aliquots of pooled plasma samples were processed the same as each individual sample to control the variation between cartridges.

#### Plasma oxylipins

Plasma arachidonic acid (ARA), eicosapentaenoic acid (EPA), docosahexaenoic acid (DHA), and oxylipins were analyzed using a liquid chromatography-multiple reaction monitoring mass spectrometry (LC-MRM-MS) method as fully described elsewhere (26). Resultant data files were processed with Skyline and the auto-integrated peaks were inspected manually. Concentrations of each oxylipin were determined from calibration curves of each analyte, which were constructed by normalizing to the selected deuterated internal standards followed by linear regression with 1/x weighting (Supplementary Data Sheet 1). The coefficient of variation for the quality control standards was <15% as reported in the method development paper (26).

#### Plasma proteome and statistical procedures

Plasma samples were centrifuged at 10,000 × *g* for 10 min to filter out particles. 5 μl of the supernatants were transferred to 96-well plates containing 55 μl of lysis buffer (8 M urea and 10 mM dithiothreitol in 50 mM triethylammonium bicarbonate). The plasma proteins were denatured and reduced by incubating at 30°C for 60 min followed by alkylation with 10 mM iodoacetamide. After dilution to reduce urea concentration to under 1 M, samples were digested with trypsin/lysine-C. The resulted peptides were acidified by adding 10 μL of 5% formic acid. After peptide concentration measurement using the standard bicinchoninic acid (BCA) assay, 200 ng peptides were loaded onto disposable EvoTip trap-columns (EV-2003, EvoSep, Denmark) and separated on an EvoSep One ™ LC system (EV-1000, EvoSep, Denmark) using a 21 min gradient with 1 μL/min flow rate. Effluents were analyzed on a high resolution Orbitrap Exploris 240 (Thermo) mass spectrometer using the data independent acquisition (DIA) method. The plasma protein library was generated using the gas-phase fractionation DIA method from peptide samples with and without depletion of the top 14 high-abundance plasma proteins (14,120 precursors, 960 proteins). For analysis of the individual samples, the range of MS1 scan was 400–1,010 m/z with 60 k orbitrap resolution and the maximum injection time was set to 50 ms with AGC target of 3 × 10^6^. The peptides were fragmented every 23 m/z scan window from 400 to 950 m/z with 1 m/z overlapping and scanned with 30 k orbitrap resolution. For the MS2 scan, maximum injection time was set to 120 ms with the automatic gain control (AGC) target of 1 × 10^6^. Pooled plasma samples were used as quality controls, injecting one per every two subject samples. The obtained LC–MS/MS dataset was searched for protein identification and quantitation using DIA-NN(27). Data were normalized by referencing to the protein levels of the first time point from the same individual subject to effectively correct for inter-individual variations (28) (Supplementary Data Sheet 2). The normalized values were statistically analyzed using the ANOVA test with two trials and six timepoints. To consider the protein as significantly changing between or within effects, the positive false discovery rate (FDR) was set to less than 0.05. Pearson-correlated hierarchical clustering analysis was used to cluster proteins with similar level patterns, and the results were visualized as a heatmap with the averaged value of each time-point after normalization by z-score. The list of significantly changed proteins in the enriched clusters were functionally enriched using STRING (Ver.11.5, https://string-db.org/). The top 10 enriched biological processes from STRING analysis were selected to represent the functions of the proteins. The STRING database does not include immunoglobulins in their analysis of protein–protein interactions. Thus, the functional enrichment analysis did not include immunoglobulins. Immunoglobulins were included in the heat map hierarchical clustering analysis, and IgM and IgG were quantified by summing all peptides belonging to the specific immunoglobulin heavy constant mu and gamma 2, respectively. Paired *t*-tests were used to compare astaxanthin and placebo trial values for IgM and IgG at each time point immediately before and after the running bout.

### Additional statistical procedures

The data are expressed as mean ± SE and were analyzed using the generalized linear model (GLM), repeated measures ANOVA module in SPSS (IBM SPSS Statistics, Version 28.0, IBM Corp, Armonk, NY, United States). The statistical model utilized the within-subjects approach: 2 (trials) × 6 (time points) repeated measures ANOVA and provided time (i.e., the collective effect of the running exercise bout) and interaction effects (i.e., whether the data pattern over time differed between trials). If the interaction effect was significant (*p* ≤ 0.05), then *post hoc* analyses were conducted using paired *t*-tests comparing time point contrasts between trials. An alpha level of *p* ≤ 0.01 was used after Bonferroni correction for five multiple tests. The positive false discovery rate (FDR or “*q* value”) was calculated for multiple testing correction of the plasma oxylipin and plasma proteomics data.

## Results

Characteristics for the *n* = 18 study participants (*n* = 11 males, *n* = 7 females) completing all aspects of the study protocol are summarized in Table 1. Male and female runners had similar ages, training histories, body compositions, and maximal oxygen consumption rates (VO_2max_). This study was not powered to compare outcome measures for the male and female runners, and outcome measures for this randomized, crossover study are presented for all participants combined.

**Table 1:** Subject characteristics (*n* = 18) for male (*n* = 11) and female (*n* = 7) runners.

	Sex	Mean	SE	*p* value
Age (years)	1 = male	40.7	2.7	0.477
	2 = female	43.7	2.9	
Body mass (kg)	1	75.4	3.8	0.008
	2	58.7	3.3	
Height (cm)	1	175.8	1.3	0.001
	2	162.8	3.7	
Body mass index (BMI) (kg/m^2^)	1	24.3	1.0	0.149
	2	22.1	0.9	
Body fat (%)	1	17.4	2.2	0.151
	2	22.9	2.9	
Maximum oxygen consumption (VO_2max_) (ml^.^kg^.-1^ min^−1^)	1	52.7	2.9	0.162
	2	46.3	2.8	
Maximum heart rate (beats/min)	1	175.2	2.3	0.062
	2	168.0	2.6	
Maximum ventilation (L/min)	1	138.7	6.6	<0.001
	2	94.3	6.9	
Maximum respiratory exchange ratio (RER_max_) (VCO_2_/VO_2_)	1	1.13	0.03	0.496
	2	1.09	0.03	
Maximum respiratory rate (breaths/min)	1	51.0	4.2	0.277
	2	44.4	3.4	
Run training distance (km/wk)	1	44.7	6.4	0.591
	2	38.8	8.9	

Three-day food records collected at the end of the 4-week supplementation period to assess the background diet revealed no significant differences in energy, carbohydrate, and micronutrient intake between trials (data not shown). For the entire group, energy intake of the background diet averaged 2,486 ± 173 kcal/day (7.24 ± 0.33 MJ/day), with carbohydrate, protein, fat, and alcohol representing 45.9 ± 1.6, 17.7 ± 1.1, 35.4 ± 1.6, and 3.0 ± 0.7%, respectively of total energy.

Performance data for each trial are summarized in Table 2. As designed, the two trials were similar in all performance measures during the first 1.75 h (level grade) including treadmill speed, total distance covered, and percent of maximal heart rates and oxygen consumption rates. During the final 30 min of the 2.25 h run, participants in the astaxanthin and placebo trials ran on a 10% downhill grade at increased speeds (11.3 ± 0.4 and 11.3 ± 0.4 km/h, respectively), similar heart rates (146 ± 2.9 and 145 ± 3.4 bpm, respectively), increased ratings of perceived exertion (RPE; 14.0 ± 0.5 and 14.3 ± 0.5, respectively), and decreased oxygen consumption rates (28.6 ± 1.3 and 28.6 ± 1.1 ml^.^kg^.-1^ min^−1^, respectively) compared to the 1.75 h level grade phase of the running bouts.

**Table 2:** Average performance outcomes for *n* = 18 runners during the first 1.75 h of the astaxanthin and placebo trials.

	Astaxanthin		Placebo		
	Mean	SE	Mean	SE	*p* value
Treadmill speed (km/h)	10.4	0.32	10.4	0.32	0.84
Heart rate (beats/min)	145	2.09	144	2.37	0.29
Heart rate (% max HR)	84.4	1.05	83.5	1.47	0.31
Total distance (2.25 h; km)	24.3	0.74	23.9	0.74	0.26
Rating perceived exertion (at 1.75 h)	13.4	0.46	13.8	0.43	0.23
Oxygen consumption (ml^.^kg^.-1^ min^−1^)	35.7	1.30	35.4	1.26	0.51
Oxygen consumption (% VO_2max_)	71.7	1.62	71.1	1.53	0.51
Ventilation (L/min)	69.9	2.46	68.0	2.76	0.10

Inflammation related data are summarized in Table 3. The 2.25 h running trials induced significant increases in delayed onset of muscle soreness (DOMS), serum concentrations for the stress hormone cortisol and the muscle damage biomarker creatine kinase, and plasma concentrations for six cytokines (all time effects, *p* ≤ 0.012). Significant post-exercise increases were also measured for serum myoglobin and the blood neutrophil-to-lymphocyte ratio (time effects, *p* < 0.001, data not shown). Interaction effects revealed no differences in the patterns of change in these biomarkers between trials.

**Table 3:** Trial comparisons of inflammation related outcomes.

Variable	Trial	Pre- study	4-week Suppl.	0 h Post-Ex	1.5 h Post-Ex	3 h Post-Ex	24 h Post-Ex	*p* value
DOMS (1–10 scale)	AS	1.4 ± 0.2	1.8 ± 0.2	5.6 ± 0.6	5.4 ± 0.5	4.9 ± 0.4	5.7 ± 0.5	<0.001; 0.818
	PL	1.5 ± 0.2	1.4 ± 0.1	5.4 ± 0.5	5.1 ± 0.5	4.5 ± 0.5	5.7 ± 0.5	
Creatine kinase (U/L)	AS	150 ± 14.9	164 ± 29.9	268 ± 35.5	265 ± 31.9	304 ± 34.2	535 ± 70.5	<0.001; 0.518
	PL	157 ± 18.6	162 ± 20.8	251 ± 29.6	262 ± 26.4	278 ± 27.8	462 ± 67.9	
Cortisol (μg/dL)	AS	15.2 ± 1.7	19.8 ± 1.4	19.9 ± 1.9	16.1 ± 2.4	15.8 ± 2.3	17.5 ± 1.0	<0.001; 0.114
	PL	16.7 ± 1.4	19.9 ± 1.2	18.3 ± 1.8	15.9 ± 1.5	13.3 ± 1.2	16.3 ± 1.4	
IL-6 (pg/mL)	AS	1.2 ± 0.1	1.7 ± 0.2	13.8 ± 2.2	9.9 ± 2.9	4.5 ± 0.5	1.8 ± 0.4	<0.001; 0.938
	PL	1.2 ± 0.2	1.9 ± 0.6	12.8 ± 2.7	8.3 ± 1.3	4.1 ± 0.4	1.8 ± 0.2	
IL-8 (pg/mL)	AS	3.3 ± 0.3	3.9 ± 0.3	8.5 ± 0.8	6.4 ± 0.6	4.6 ± 0.4	3.7 ± 0.3	<0.001; 0.123
	PL	3.6 ± 0.3	4.0 ± 0.4	7.7 ± 0.7	6.3 ± 0.5	4.4 ± 0.3	3.6 ± 0.3	
IL-10 (pg/mL)	AS	2.3 ± 0.5	3.7 ± 1.2	17.7 ± 5.4	9.1 ± 3.0	4.2 ± 0.9	2.9 ± 0.6	0.012; 0.079
	PL	2.4 ± 0.5	2.8 ± 0.6	12.4 ± 4.4	5.4 ± 1.5	2.7 ± 0.5	3.0 ± 0.5	
IL-1ra (pg/mL)	AS	119 ± 4.9	143 ± 12.8	244 ± 29.6	283 ± 38.3	291 ± 33.0	146 ± 11.4	<0.001; 0.225
	PL	122 ± 6.4	134 ± 6.9	182 ± 11.8	243 ± 28.0	244 ± 24.3	137 ± 7.6	
MCP-1 (pg/mL)	AS	149 ± 9.0	161 ± 11.9	292 ± 23.6	260 ± 20.6	216 ± 12.6	154 ± 9.3	<0.001; 0.123
	PL	153 ± 10.9	164 ± 9.2	261 ± 21.7	258 ± 16.8	213 ± 16.3	165 ± 8.3	
GCSF (pg/mL)	AS	12.7 ± 1.8	12.5 ± 1.9	20.4 ± 2.4	23.9 ± 5.5	18.3 ± 3.5	15.6 ± 3.4	<0.001; 0.565
	PL	11.4 ± 1.9	13.2 ± 2.0	19.3 ± 2.4	20.3 ± 2.4	15.9 ± 2.6	12.7 ± 2.0	

AS, astaxanthin; PL, placebo. *p* values represent time (first value) and trial × time interaction effects. All interaction effects were non-significant, so no post-hoc tests were conducted.

**Figure 3:**
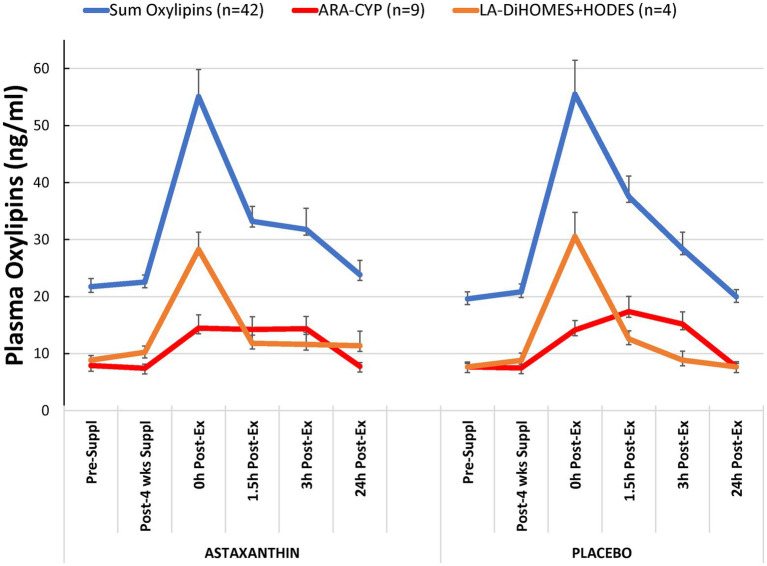
Plasma oxylipin concentrations for the astaxanthin and placebo trials. “Sum oxylipins” is the sum value for *n* = 42 oxylipins with a significant exercise-induced time effect but no interaction effect (*p* = 0.412). “ARA-CYP” is the sum value for *n* = 9 arachidonic acid-cytochrome P-450 generated oxylipins (time effect, *p* < 0.001, interaction effect, *p* = 0.432). “LA-DiHOMES+HODES” is the sum value for *n* = 4 linoleic acid CYP and LOX generated oxylipins (time effect, *p* < 0.001, interaction effect, *p* = 0.505).

Of 81 oxylipins detected in study samples, a total of 42 oxylipins exhibited significant time effects during GLM statistical analysis (Supplementary Data Sheet 1). These 42 oxylipins were summed for a composite variable (Figure 3). GLM analysis showed a significant time effect (*p* < 0.001) of 2.25 h running on this composite variable of 42 oxylipins, but without trial differences (interaction effect, *p* = 0.412) or differences between male and female runners (supplement × time × sex interaction effect, value of *p* = 0.800). Two other composite variables were calculated including nine oxylipins generated from arachidonic acid and cytochrome P-450 (ARA-CYP) and four abundant oxylipins generated from linoleic acid and CYP (9,10-DiHOME, 12,13-DiHOME) and lipoxygenase (LOX; 9-HODE, 13-HODE; LA-DiHOMES+HODES; Figure 3). Significant time effects were shown for ARA-CYP and LA-DiHOMES+HODES (*p* = 0.007 and *p* < 0.001, respectively), but without trial differences (*p* = 0.432 and *p* = 0.505, respectively). The nine oxylipins included with ARA-CYP are generally regarded as pro-inflammatory oxylipins and included 5,6-, 8,9-, 11,12-, and 14,15-diHETrEs, 5,15-diHETE, 16-, 17,- 18-HETEs, and the 20-HETE metabolite 20-coohAA.

A total of 608 plasma proteins were identified with an FDR <0.01, and 500 of them were quantified without any missing values across 214 samples analyzed (Supplementary Data Sheet 2). The plasma sample quality was assessed based on the plasma’s potential main sources of protein contamination, indicating that the majority of samples (all but three) were not significantly contaminated with platelets, erythrocytes, or coagulations (Supplementary Figure S1A). Since the protein levels varied significantly between subjects as evidenced by the higher correlations within subjects than between subjects (Supplementary Figure S1B), the longitudinal dataset was normalized by calculating ratios to the protein levels at the first time point to increase the likelihood of discovering proteins dysregulated due to supplementation.

**Figure 4:**
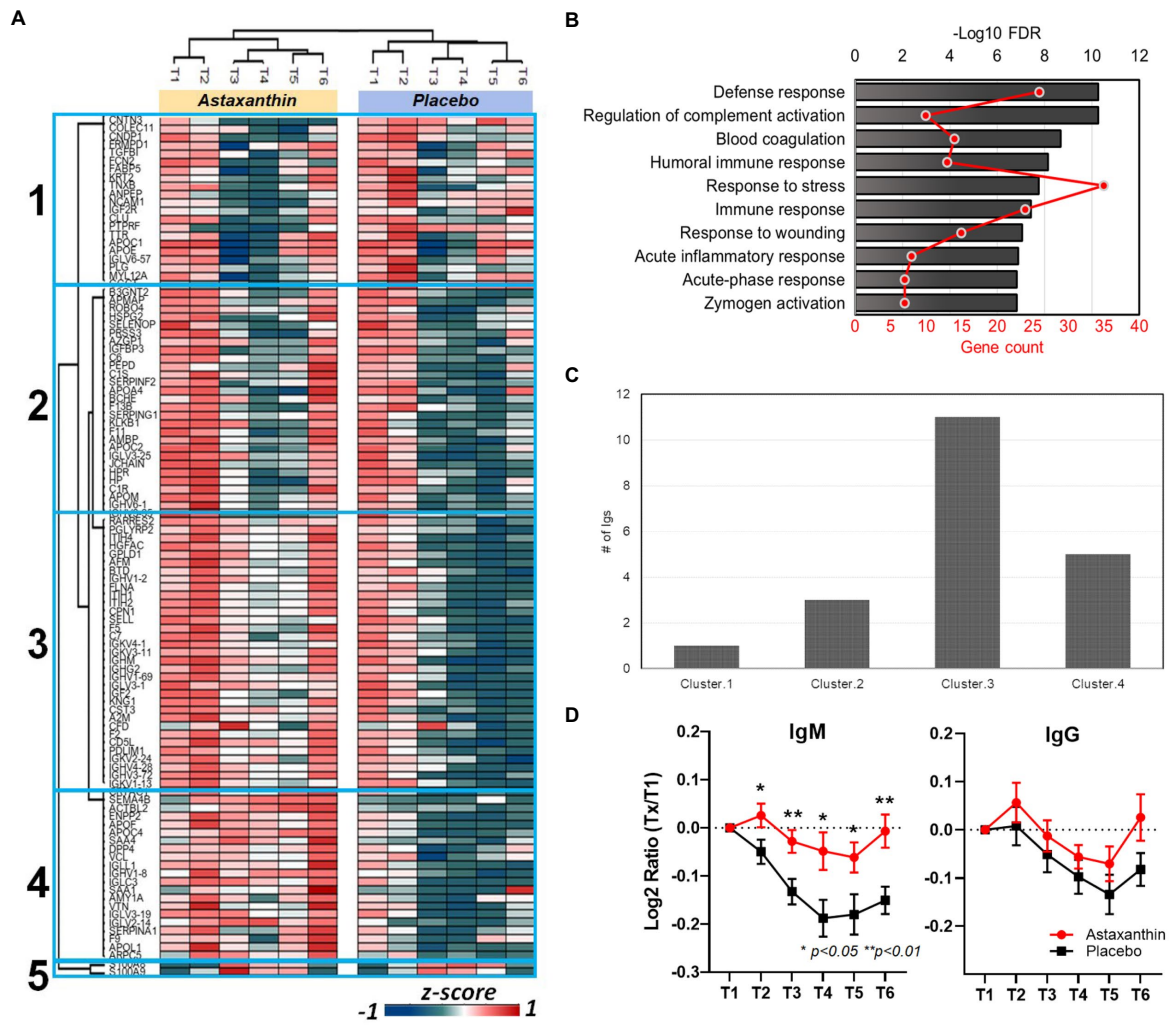
**(A)** Heatmap of clustered proteins in the astaxanthin and placebo trials. T1, pre-study; T2, 4-weeks supplementation, pre-exercise; T3, immediately post-exercise (2.25 h run bout); T4, 1.5 h post-exercise; T5, 3 h post-exercise; T6, 24 h post-exercise. **(B)** Associated biological processes for clusters 2–4, see Supplementary Table S1 for details. **(C)** Number of identified immunoglobulins in the clusters of **(A)**. **(D)** Changes of plasma IgM and IgG levels in plasma in subjects in response to the astaxanthin and placebo trials.

Of the 500 identified plasma proteins, 105 were significantly influenced by the running bouts (FDR < 0.05, Figure 4A). Cluster 1 proteins (23 total) were rapidly downregulated after exercise and then gradually recovered in both supplement trials within 24 h. A total of 82 proteins from clusters 2, 3, and 4 (Figure 4A) were immediately reduced post-exercise compared to pre-exercise levels and increased during the 24 h post-exercise period in the astaxanthin compared to the placebo trial. Biological process analysis revealed that most of the proteins were involved in immune-related functions such as defense responses, complement activation, and immune system responses (Figure 4B; Table 4). Two proteins in cluster 5, S100A8 and S100A9, were increased after exercise in both groups and then gradually returned to pre-exercise levels within the 24 h post-exercise recovery period. Their biological functions are related to neutrophil degranulation (FDR = 0.002) and the innate immune system (FDR = 0.0095). A total of 20 plasma immunoglobulins were identified that differed significantly between the astaxanthin and placebo trials (Figure 4C). Plasma levels of IgM were significantly downregulated post-exercise but recovered after the 24 h post-exercise recovery period in the astaxanthin but not the placebo trial (Figure 4D). The patterns of change in IgG did not differ between the astaxanthin and placebo trials.

**Table 4:** Associated biological processes, gene counts, and matching proteins for clusters 2–4.

Biological processes	FDR *q* value	Gene count	Matching proteins, clusters 2,3,4
Defense response	5.51E-11	26	IGJ, C6, KLKB1, KNG1, ITIH4, SAA4, SERPING1, F2, APOL1, SERPINF2, C7, IGLL1, CFD, PGLYRP2, HP, APOA4, DPP4, CD5L, FLNA, HSPG2, CST3, SAA1, C1S, SERPINA1, RARRES2, and C1R
Regulation of complement activation	5.51E-11	10	VTN, C6, SERPING1, F2, C7, A2M, CD5L, CPN1, C1S, and C1R
Blood coagulation	2.08E-09	14	VCL, F9,KLKB1, KNG1, SERPING1, F2,A2M, F13B, F5, FLNA, SAA1,F11, SERPINA1, and HGFAC
Humoral immune response	7.39E-09	13	IGJ, C6, KNG1, SERPING1, F2, C7, IGLL1, CFD, PGLYRP2, PRSS3, C1S, RARRES2, and C1R
Response to stress	1.76E-08	35	VCL, F9, IGJ, C6,KLKB1, KNG1, ITIH4, SAA4, SERPING1, F2, APOL1, SERPINF2, C7, A2M, IGLL1, CFD, PGLYRP2, HP, APOA4, DPP4, F13B, F5, CD5L, FLNA, PDLIM1, HSPG2, CST3, SAA1, F11, C1S, SERPINA1, RARRES2, SEPP1, HGFAC, and C1R
Immune response	3.83E-08	24	VCL, VTN, SELL, IGJ, ENPP2, C6, KNG1, SERPING1, ARPC5, F2, APOL1, C7, IGLL1, CFD, PGLYRP2, HP, APOA4, PRSS3, CST3, SAA1, C1S, SERPINA1, RARRES2, and C1R
Response to wounding	8.98E-08	15	VCL, F9, KLKB1, KNG1, SERPING1, F2, A2M, F13B, F5, FLNA, CST3, SAA1, F11, SERPINA1, and HGFAC
Acute inflammatory response	1.29E-07	8	KLKB1, ITIH4, SAA4, F2, SERPINF2, HP, SAA1, and SERPINA1
Acute-phase response	1.51E-07	7	ITIH4, SAA4, F2, SERPINF2, HP, SAA1, and SERPINA1
Zymogen activation	1.51E-07	7	F9, KLKB1, PRSS3, CD5L, F11, HGFAC, and C1R

## Discussion

This study employed a strong research design and showed that 4-weeks astaxanthin supplementation had no effect in runners on 2.25-h running-induced muscle soreness, muscle damage, and elevations in six plasma cytokines and 42 oxylipins. The untargeted proteomics data, however, showed that astaxanthin supplementation did counter exercise-induced decreases in 82 plasma proteins involved in immune-related functions. Astaxanthin supplementation countered the post-exercise decrease in plasma immunoglobulins, especially IgM.

Other astaxanthin-based human clinical trials focused on limited and basic outcomes related to exercise performance, muscle damage (e.g., creatine kinase), oxidative stress (e.g., malondialdehyde, MDA, as a lipid peroxidation marker), and inflammation (e.g., C-reactive protein, CRP (15–20, 29). Most of these studies showed no effects of astaxanthin (6–20 mg/day during 1–13 weeks) on exercise performance (29), creatine kinase (17, 18), MDA (18, 29), or CRP (17, 18) after an exercise challenge. This is the first human clinical trial to measure physiological responses to astaxanthin supplementation after an intense exercise challenge using untargeted proteomics (500 proteins across all samples), a targeted and comprehensive panel of 81 oxylipins, and six cytokines.

The data indicate that 4-weeks astaxanthin supplementation had little effect on exercise-induced increases in most inflammation-related measures including six plasma cytokines, 42 plasma oxylipins, and plasma proteins in cluster 5 of this study. The running bout caused significant increases in plasma levels of IL-6, IL-8, IL-10, MCP-1, GCSF, IL1ra, and many different types of oxylipins as shown in previous studies (3, 5, 6, 21). The proteomics analysis showed that two proteins in cluster 5, S100A8 and S100A9 or calprotectin, were increased after exercise in both the astaxanthin and placebo trials, before gradually returning to pre-exercise levels within the 24 h post-exercise recovery period. Calprotectin is released during degranulation from activated neutrophils during the inflammatory process following intensive exercise. Calprotectin also promotes phagocyte migration, and functions as an alarmin and endogenous danger-associated molecular pattern (DAMP) (30). *In vivo* and *in vitro* data support a role for astaxanthin in decreasing inflammation, but the data from the present study indicate that these findings do not extend to mitigating transient exercise-induced inflammation (9, 17, 18).

Astaxanthin supplementation did have a strong effect in countering post-exercise decreases in many proteins related to immune function including 20 immunoglobulins. The major soluble proteins for humoral immunity are the immunoglobulins that can combine with specific antigens as a functional component of the host defense system. Previous studies have shown that serum immunoglobulin levels can be reduced for 1–2 days after prolonged and intensive exercise, as confirmed in the present study (31, 32). B lymphocyte suppression has been reported after sustained vigorous exercise and may in part be related to an inhibitory effect from activated monocytes (33). Several cell culture-based studies have shown that astaxanthin can increase immunoglobulin production under varying conditions (14, 27, 34–36). For example, astaxanthin enhanced IgM and IgG production by human lymphocytes in response to T cell-dependent stimuli (14). Animal studies support increases in plasma IgG and IgM and other biomarkers of immune function in astaxanthin-fed dogs and cats (37, 38). In the present study, astaxanthin supplementation countered the exercise-induced decrease in plasma IgM but not IgG levels. IgM represents about 10% of total blood immunoglobulins and is the predominant antibody produced early in an immune response. IgM is also the major immunoglobulin expressed on the surface of B cells (31).

Randomized clinical trials investigating the influence of astaxanthin supplementation on immune-related outcomes are limited. One study of young female adults ingesting 0, 2, or 8 mg/day astaxanthin daily for 8 weeks showing strong increases in plasma astaxanthin concentrations at 4 and 8 weeks with 2 or 8 mg/day, but inconsistent and modest improvements in T cell and natural killer cell function (13). Plasma immunoglobulins were not measured in this study. The data from the present study are the first human data to indicate that astaxanthin supplementation can counter exercise-induced decreases in plasma immunoglobulins and IgM in human subjects.

## Conclusion

This study used a 2.25 h running bout with 30 min of downhill running to induce muscle soreness, damage, inflammation, and immune dysfunction. Astaxanthin supplementation (8 mg/day, 4 weeks) was tested under double blind procedures against placebo using a crossover design. The objective was to see if astaxanthin could serve as a nutrition-based strategy to mitigate exercise-induced physiological stress. A human systems biology approach was used to improve the ability to capture trial differences using untargeted proteomics, and comprehensive targeted oxylipin and cytokine panels. These data indicate that astaxanthin supplementation did not counter exercise-induced increases in plasma cytokines and oxylipins but was linked to normalization of post-exercise plasma levels of numerous immune-related proteins within 24 h. Thus, astaxanthin supplementation provided immune support for runners engaging in a vigorous running bout and uniquely countered decreases in 20 plasma immunoglobulins including IgM.

## Data availability statement

The mass spectrometry proteomics data have been deposited to the ProteomeXchange Consortium via the PRIDE partner repository with the dataset identifier PXD040503.

## Ethics statement

The studies involving human participants were reviewed and approved by Appalachian State University IRB. The patients/participants provided their written informed consent to participate in this study.

## Author contributions

DN, QZ, AP, and GV designed the research project. DN, CS, KD, AP, and GV conducted the research project. JW, QZ, AO, YT, CS, and KD analyzed the samples, and DN, JW, and QZ conducted the data analysis. DN, JW, QZ, CS, KD, AO, AP, GV, and YT wrote and edited the paper. DN had primary responsibility for the final content. All authors contributed to the article and approved the submitted version.

## Funding

The authors declare that this study received funding from Lycored. The funder was not involved in the study design, collection, analysis, interpretation of data, the writing of this article, or the decision to submit it for publication.

## Conflict of interest

The authors declare that the research was conducted in the absence of any commercial or financial relationships that could be construed as a potential conflict of interest.

## Publisher’s note

All claims expressed in this article are solely those of the authors and do not necessarily represent those of their affiliated organizations, or those of the publisher, the editors and the reviewers. Any product that may be evaluated in this article, or claim that may be made by its manufacturer, is not guaranteed or endorsed by the publisher.
