# Supplementary figures and images for: Astaxanthin supplementation counters exercise-induced decreases in immune-related plasma proteins
Source: Front Nutr. 2023 Mar 21;10:1143385. doi: 10.3389/fnut.2023.1143385 (PMC10070989; doi:10.3389/fnut.2023.1143385)

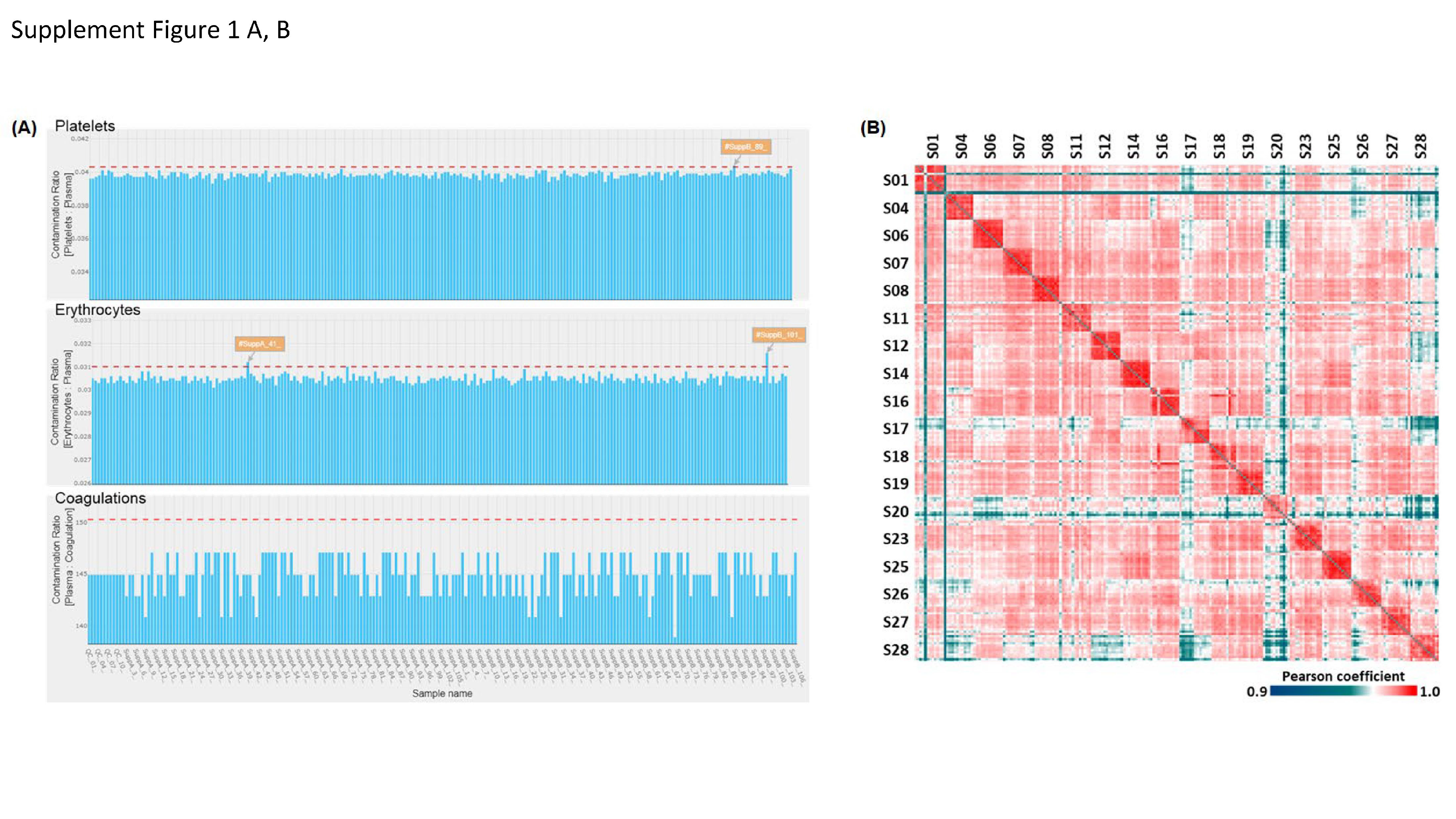

Supplement: Supplementary file 3 [file Image_1.TIFF]
